# ALS With and Without Upper Motor Neuron Signs: A Comparative Study Supporting the Gold Coast Criteria

**DOI:** 10.1002/acn3.70288

**Published:** 2025-12-15

**Authors:** Hee‐Jae Jung, E‐nae Cheong, Jungmin So, Heung‐Won Kang, Yangsean Choi, Eun‐Jae Lee, Hyunjin Kim, Young‐Min Lim

**Affiliations:** ^1^ Department of Neurology, Asan Medical Center University of Ulsan College of Medicine Seoul Republic of Korea; ^2^ Department of Neurology Seoul Medical Center Seoul Republic of Korea; ^3^ Department of Radiology and Research Institute of Radiology, Asan Medical Center University of Ulsan College of Medicine Seoul Republic of Korea; ^4^ Department of Neurology Korea University Ansan Hospital Ansan Republic of Korea; ^5^ Department of Medical Science, Asan Medical Center University of Ulsan College of Medicine Seoul Republic of Korea

**Keywords:** amyotrophic lateral sclerosis, Gold Coast criteria, upper motor neuron

## Abstract

**Objective:**

The Gold Coast criteria permit diagnosis of amyotrophic lateral sclerosis (ALS) even without upper motor neuron (UMN) signs. However, whether ALS patients with UMN signs (ALSwUMN) and those without (ALSwoUMN) share similar characteristics and prognoses remains unclear. This study compared clinical features, disease progression, electrophysiological findings, biomarker profiles, imaging parameters, and survival between these groups.

**Methods:**

ALS patients diagnosed according to the Gold Coast criteria were classified into ALSwUMN (*n* = 51) and ALSwoUMN (*n* = 20) groups. We evaluated clinical data, motor evoked potentials (MEP), and serum biomarkers, including cardiac Troponin T, neurofilament light chain, glial fibrillary acidic protein, and brain‐derived neurotrophic factor. Imaging parameters, including cortical thickness and white matter volume, were also evaluated. Survival was analyzed using the Kaplan–Meier method.

**Results:**

The groups showed broadly similar clinical features, disease progression, and biomarker profiles. Abnormal MEPs were more frequent in ALSwUMN (94.0%) than in ALSwoUMN (63.2%, *p* = 0.017). Both groups demonstrated cortical thinning in the precentral and entorhinal regions compared to healthy controls. ALSwUMN exhibited thinning in the lateral orbitofrontal, insular, and temporal pole regions, while ALSwoUMN showed thinning in the pars opercularis. White matter volume was reduced in both groups in the thalamus, cerebellum, and amygdala, with additional brainstem atrophy in ALSwUMN. No significant survival difference was observed.

**Interpretation:**

Despite minor distinctions in electrophysiological and imaging findings, ALSwoUMN had overall comparable clinical profiles and outcomes to ALSwUMN. These findings support recognizing ALSwoUMN within the ALS spectrum under the Gold Coast criteria.

## 1 Introduction

Amyotrophic lateral sclerosis (ALS) is a progressive neurodegenerative disease characterized by degeneration of motor neurons in the brain and spinal cord, ultimately leading to muscle weakness and atrophy [1]. Traditionally, a diagnosis of ALS requires the presence of both upper motor neuron (UMN) and lower motor neuron (LMN) involvement [2, 3]. Although UMN signs such as hyperreflexia and spasticity are identifiable on physical examination, their detection can be challenging, particularly in the early stages of the disease or in the presence of profound LMN loss [4]. This diagnostic uncertainty complicates the differentiation of ALS from phenotypically similar disorders, such as progressive muscular atrophy (PMA) or other LMN syndromes [5, 6].

To address these challenges, the Gold Coast criteria were introduced in 2020 to simplify ALS diagnosis and improve sensitivity [7, 8, 9, 10]. These criteria allow ALS to be diagnosed in the absence of UMN signs, provided LMN involvement is present in at least two regions and the disease exhibits progressive clinical deterioration [2]. This represents a major shift from previous diagnostic frameworks, such as the revised El Escorial and Awaji criteria, which required clinical evidence of UMN dysfunction [2, 3].

Despite the inclusive nature of the Gold Coast criteria, whether ALS patients without observable UMN signs (ALSwoUMN) represent the same disease spectrum as those with UMN signs (ALSwUMN) remains uncertain. Prior studies suggest that PMA patients—clinically equivalent to ALSwoUMN—may have a more favorable median survival (approximately 48 months) than classic ALS (approximately 36 months). However, survival curves tend to converge beyond 80 months, and the later appearance of UMN signs does not seem to significantly alter prognosis [11, 12, 13]. Nevertheless, autopsy and imaging studies frequently reveal underlying UMN pathology in such patients [14, 15]. Validating the Gold Coast criteria therefore requires direct comparisons of ALSwUMN and ALSwoUMN to clarify similarities and differences in clinical features, neurophysiology, neuroimaging, biomarkers, and survival outcomes.

This study aims to address this knowledge gap by comparing ALSwUMN and ALSwoUMN diagnosed according to the Gold Coast criteria. We investigate their clinical profiles, electrophysiological features, structural MRI–based cortical thickness and white matter volume changes, serum biomarkers, and survival to determine whether these phenotypes align within a unified ALS spectrum and to provide in vivo insights into corticospinal and cortical degeneration across the ALS continuum.

## 2 Subjects/Materials and Methods

### 2.1 Study Participants

Patients were consecutively and prospectively recruited at Asan Medical Center, a tertiary referral hospital in Seoul, Republic of Korea, between April 2022 and June 2023. The inclusion criteria were: (1) a new diagnosis of ALS according to the Gold Coast criteria [7], and (2) age 18 years or older. The exclusion criteria were: (1) a known history of other major neuropsychiatric diseases, such as Parkinson's disease, Alzheimer's disease, epilepsy, schizophrenia, or major depressive disorder; (2) severe systemic illnesses unrelated to ALS that could interfere with study procedures, such as advanced cardiovascular disease or active malignancy; and (3) any evidence of other central or peripheral nervous system disorders that could mimic ALS. All eligible patients were invited to participate, and those who provided written informed consent were enrolled.

Enrolled patients were then classified into two groups based on the presence of clinical upper motor neuron (UMN) signs at diagnosis: (1) ALSwUMN: those exhibiting both UMN and lower motor neuron (LMN) signs in at least one body region, with both present in the same region if only one region was affected; and (2) ALSwoUMN: those showing LMN involvement in at least two regions but lacking any clinical UMN signs.

For imaging analyses, a control group of 80 age‐ and sex‐matched individuals without neurological disease or abnormal MRI findings was recruited at the same institution during the same period and used as the reference cohort.

The study protocol was approved by the Institutional Review Board of Asan Medical Center (IRB No. 2023–1064) and conducted in accordance with the Declaration of Helsinki.

### 2.2 Clinical Data Collection

Demographic and clinical information was collected at enrollment, including age at symptom onset, sex, site of symptom onset (bulbar or limb), side of limb onset, and disease duration from symptom onset to study inclusion. Clinical UMN involvement was defined as the presence of one or more of the following on neurological examination: pathologically brisk deep tendon reflexes (biceps, triceps, knee, or ankle jerks), brisk jaw jerk, pseudobulbar palsy, Hoffmann's sign, Babinski's sign (extensor plantar response), ankle clonus, or spasticity characterized by a velocity‐dependent increase in muscle tone. Neurological examinations were performed by experienced neuromuscular neurologists (H‐J. J. or J. S.). Functional status was assessed using the ALS Functional Rating Scale–Revised (ALSFRS‐R), with subscores calculated for bulbar, limb, and respiratory domains. The disease progression rate was calculated as: (48 − ALSFRS‐R at enrollment)/disease duration in months [16]. Patients were categorized as slow or fast progressors using a cutoff of 0.9 points/month (≤ 0.9 vs. > 0.9) [17].

### 2.3 Motor Evoked Potential

Transcranial magnetic stimulation (TMS) was performed using a Magstim Rapid [2] stimulator with a circular coil. Motor evoked potentials (MEPs) were recorded bilaterally from the abductor digiti quinti (ADQ) muscles in the upper limbs and the tibialis anterior (TA) muscles in the lower limbs. Both sides were examined. Central motor conduction time (CMCT) was calculated by subtracting the peripheral motor conduction time (latency of the MEP elicited by cervical or lumbar root stimulation) from the total motor conduction time (latency of the MEP elicited by cortical stimulation). MEPs were considered abnormal if either of the following criteria was met: (1) absence of a cortical MEP despite a recordable peripheral CMAP, or (2) a CMCT exceeding the mean by more than 2.5 standard deviations of reference values. Normative CMCT values were obtained from Clinical Electromyography, 3rd edition [18].

### 2.4 Pulmonary Function Testing

Upright forced vital capacity (FVC) was assessed using a spirometer. The highest value from three reproducible attempts was recorded as the FVC (% predicted), serving as a surrogate marker of respiratory muscle strength [19].

### 2.5 Serum Biomarker Analysis

Peripheral venous blood samples were collected at baseline and centrifuged at 2000 *g* for 15 min at 4°C within 1 h. Serum aliquots were stored at −80°C until analysis. Concentrations of neurofilament light chain (NfL), glial fibrillary acidic protein (GFAP), and brain‐derived neurotrophic factor (BDNF) were measured using the Simoa HD‐1 Analyzer (Quanterix, MA, USA) with commercially available kits according to standardized protocols [20]. All assays were performed in duplicate, and operators were blinded to clinical data. All measurements exceeded the lower limit of quantification after appropriate dilution (0.241 pg/mL for NfL, 0.467 pg/mL for GFAP, and 0.0293 pg/mL for BDNF). The mean intra‐assay coefficients of variation were 3.9% for NfL, 3.2% for GFAP, and 3.2% for BDNF. Duplicate measurements varied by less than 20%. Serum cardiac Troponin T (cTnT) was measured using electrochemiluminescence immunoassay (ECLIA) with the Elecsys Troponin‐T hs assay (Roche Diagnostics, Basel, Switzerland). Serum creatinine was measured using the enzymatic colorimetric method in the hospital's certified laboratory, with a reference interval of 0.7–1.4 mg/dL for adults.

### 2.6 MRI Acquisition and Analysis

All brain MRI scans were acquired using a 3.0‐T scanner (Ingenia CX, Philips Medical Systems, Netherlands) with a 32‐channel head coil. High‐resolution 3D T1‐weighted images were obtained (repetition time [TR] = 6.5 ms; echo time [TE] = 2.9 ms; flip angle [FA] = 9°; field of view [FOV] = 256 × 256 mm^2^; voxel size = 1 × 1 × 1 mm^3^). Additional sequences included 2D T2 FLAIR (TR = 9000 ms; TE = 125 ms; FA = 90°; FOV = 220 × 220 mm^2^; voxel size = 0.8 × 0.8 × 3 mm^3^), 2D T2‐weighted (TR = 3000 ms; TE = 80 ms; FA = 90°; FOV = 384 × 384 mm^2^; voxel size = 0.6 × 0.6 × 4 mm^3^), and susceptibility‐weighted imaging (TR = 31 ms; TE = 7.2 ms; FA = 17°; FOV = 368 × 297 mm^2^; voxel size = 0.5 × 0.5 × 2 mm^3^).

Cortical thickness analysis was performed using FreeSurfer v7.3, with preprocessing steps including skull stripping, surface inflation, segmentation, and registration to the Desikan–Killiany atlas. White matter (WM) volume was quantified using the SPM12 toolbox in MATLAB, with DARTEL normalization and modulation applied. Regional volumes were normalized to intracranial volume. Manual quality control was applied to all segmentations [21, 22, 23]. Detailed image processing steps are provided in the Supporting Information.

### 2.7 Survival Analysis

Survival was defined as the interval from baseline to death. For patients alive at the end of follow‐up (January 2025), survival time was censored at the date of the last available clinic visit. Kaplan–Meier curves were generated and compared using the log‐rank test. Cox proportional hazards regression models were adjusted for age, sex, site of onset, ALSFRS‐R score, UMN group status, and progression rate.

### 2.8 Statistical Analysis

Continuous variables were compared using *t*‐tests or Mann–Whitney *U* tests, depending on data distribution. Categorical variables were analyzed using chi‐square tests. Differences in cortical thickness and regional WM volume were tested using two‐sample t‐tests with false discovery rate (FDR) correction for multiple comparisons. Statistical analyses were performed using R (v3.4.1) and SPSS (v25). A two‐tailed *p*‐value < 0.05 was considered statistically significant.

## 3 Results

### 3.1 Study Population

**FIGURE 1:**
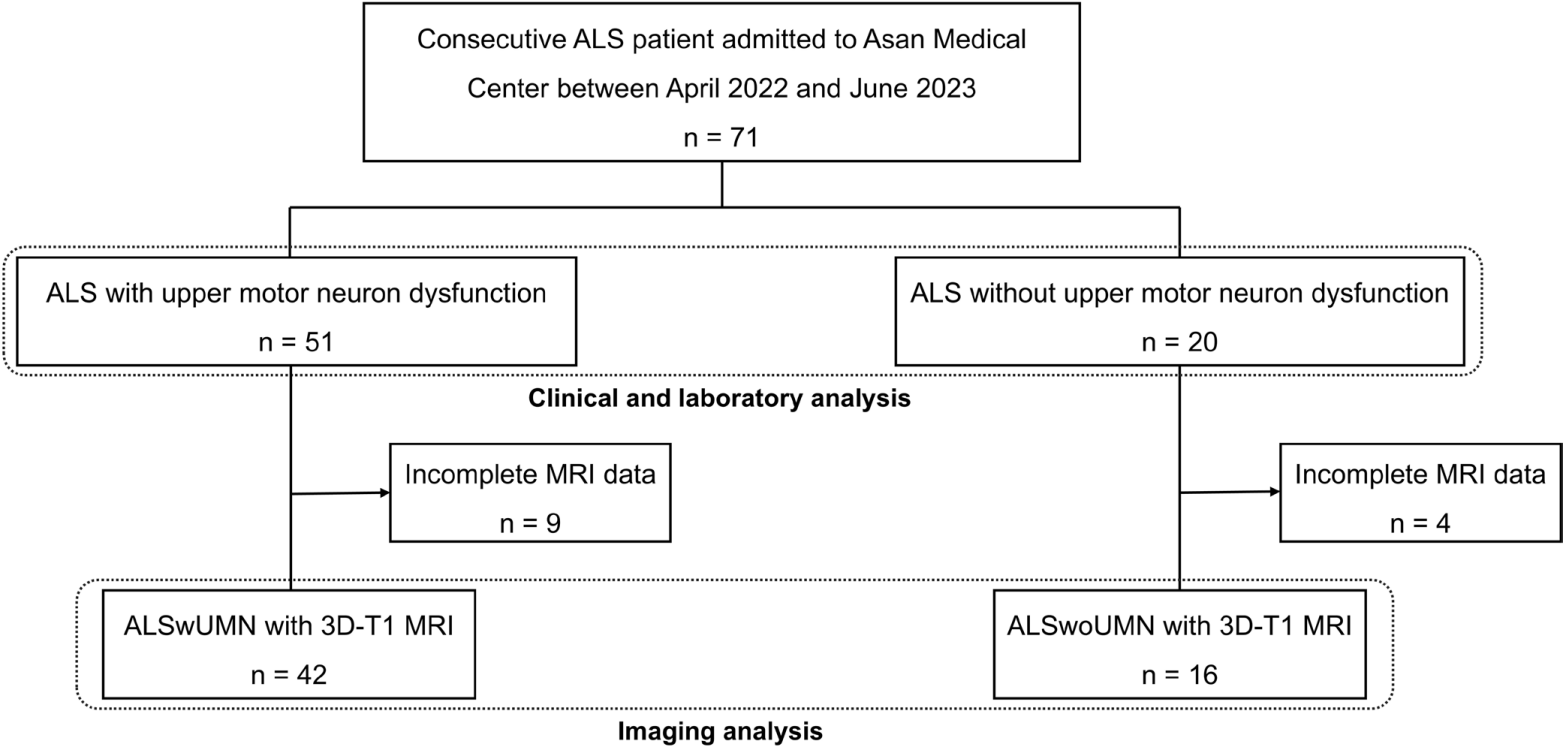
Study flow chart. 3D‐T1 MRI, three‐dimensional T1‐weighted magnetic resonance imaging; ALS, amyotrophic lateral sclerosis; ALSwoUMN, ALS patients without upper motor neuron signs; ALSwUMN, ALS patients with upper motor neuron signs.

A total of 71 patients diagnosed with ALS according to the Gold Coast criteria were enrolled, including 51 in the ALSwUMN group and 20 in the ALSwoUMN group. All patients had sporadic ALS. During follow‐up, two patients initially classified as ALSwoUMN later developed overt UMN signs. For MRI analysis, 13 patients (9 ALSwUMN and 4 ALSwoUMN) were excluded due to incomplete imaging, leaving 58 patients with evaluable MRI data (42 ALSwUMN and 16 ALSwoUMN; Figure 1). Detailed information regarding the characteristics of the control group is provided in Table S1.

### 3.2 Baseline Clinical Characteristics

Table 1 summarizes the baseline characteristics of both groups. The mean age at enrollment was 66.2 ± 10.4 years, with no significant difference between ALSwUMN and ALSwoUMN groups (65.0 ± 10.5 vs. 69.4 ± 10.0 years, *p* = 0.115). The mean age at symptom onset was also comparable (62.3 ± 10.6 vs. 66.5 ± 9.7 years, *p* = 0.136). The average disease duration from symptom onset to enrollment was 19.1 ± 12.8 months, with no significant group difference (*p* = 0.482). Males comprised 57.7% of the cohort, with similar distributions in ALSwUMN (54.9%) and ALSwoUMN (65.0%) groups (*p* = 0.446). Regarding symptom onset, 32.4% of patients had bulbar onset, 45.1% had cervical onset, and 22.5% had lumbar onset. The distribution did not differ significantly between groups (*p* = 0.549). The mean total ALSFRS‐R score was 36.1 ± 8.5, with ALSwUMN scoring slightly higher than ALSwoUMN (36.7 ± 7.4 vs. 34.6 ± 10.8, *p* = 0.347), though the difference was not statistically significant. Subdomain ALSFRS‐R scores (bulbar, upper extremity, lower extremity, and respiratory) were also comparable (all *p* > 0.2). The disease progression rate, defined as monthly decline in ALSFRS‐R score, was 0.9 ± 1.1/month overall, with no significant difference between ALSwUMN and ALSwoUMN (0.9 ± 1.1 vs. 0.8 ± 0.9, *p* = 0.765). Patients were classified as slow or fast progressors based on this rate, with 64.8% categorized as slow and 35.2% as fast progressors. The distribution of progression types did not differ significantly between groups (*p* = 0.266).

**TABLE 1:** Clinical characteristics of patients with amyotrophic lateral sclerosis.

	Total	ALSwUMN	ALSwoUMN	*p*
	(*n* = 71)	(*n* = 51)	(*n* = 20)	
Age at enrollment, years	66.2 ± 10.4	65.0 ± 10.5	69.4 ± 10.0	0.115
Age at onset, years	63.5 ± 10.4	62.3 ± 10.6	66.5 ± 9.7	0.136
Disease duration, months	19.1 ± 12.8	18.5 ± 13.0	20.9 ± 12.6	0.482
Male	41 (57.7%)	28 (54.9%)	13 (65.0%)	0.446
Onset site				0.549
Bulbar	23 (32.4%)	15 (29.4%)	8 (40.0%)	
Cervical	32 (45.1%)	25 (49.0%)	7 (35.0%)	
Lumbar	16 (22.5%)	11 (21.6%)	5 (25.0%)	
Onset side				0.451
Right	13 (18.3%)	9 (17.6%)	4 (20.0%)	
Left	18 (25.4%)	15 (29.4%)	3 (15.0%)	
Undetermined	40 (56.3%)	27 (52.9%)	13 (65.0%)	
ALSFRS‐R score				
Total	36.1 ± 8.5	36.7 ± 7.4	34.6 ± 10.8	0.347
Bulbar	9.4 ± 2.7	9.5 ± 2.8	9.4 ± 2.8	0.869
Upper extremity	8.1 ± 3.4	8.4 ± 3.1	7.3 ± 3.9	0.209
Lower extremity	8.2 ± 3.5	8.3 ± 3.4	8.0 ± 3.8	0.727
Respiratory	10.4 ± 3.0	10.6 ± 2.8	10.0 ± 3.7	0.498
Progression rate, /month	0.9 ± 1.1	0.9 ± 1.1	0.8 ± 0.9	0.765
Progression type				0.266
Slow (progression rate ≤ 0.9)	46 (64.8)	31 (60.8)	15 (75.0)	
Fast (progression rate > 0.9)	25 (35.2)	20 (39.2)	5 (25.0)	

Abbreviations: ALS, amyotrophic lateral sclerosis; ALSFRS‐R, ALS functional rating scale‐revised; ALSwoUMN, ALS patients without upper motor neuron signs; ALSwUMN, ALS patients with upper motor neuron signs.

### 3.3 Electrophysiological and Laboratory Findings

**FIGURE 2:**
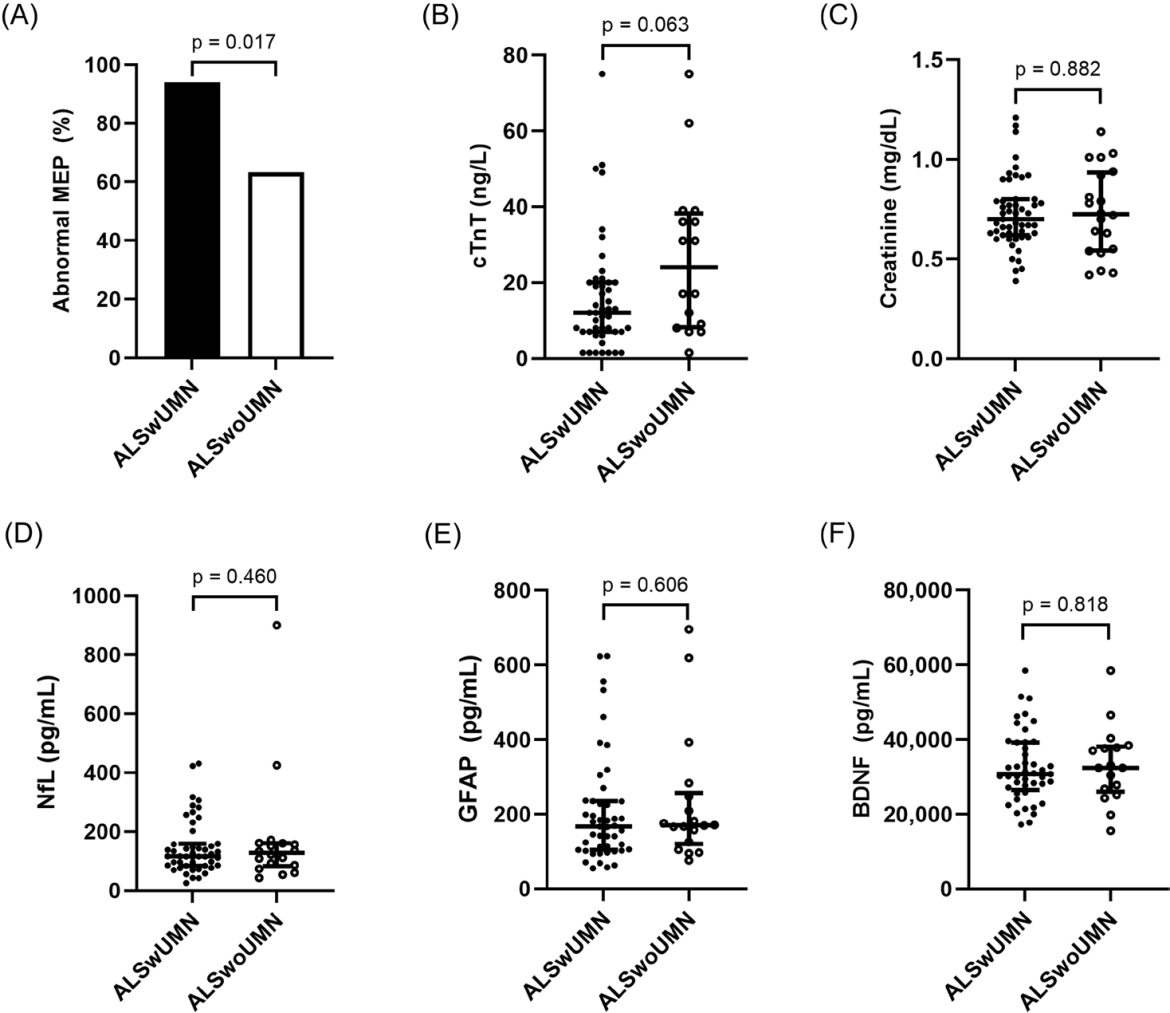
Comparison of laboratory characteristics between ALSwUMN and ALSwoUMN groups. Panels show results for (A) motor evoked potential (MEP), (B) cardiac Troponin T (TnT), (C) serum creatinine, (D) neurofilament light chain (NfL), (E) glial fibrillary acidic protein (GFAP), and (F) brain‐derived neurotrophic factor (BDNF). ALS, amyotrophic lateral sclerosis; ALSwoUMN, ALS patients without upper motor neuron sign; ALSwUMN, ALS patients with upper motor neuron sign.

As illustrated in Figure 2, abnormal MEPs were significantly more frequent in ALSwUMN (94.0%, 47/50) than in ALSwoUMN (63.2%, 12/19; *p* = 0.017). This indicates greater electrophysiological UMN involvement in ALSwUMN, though subclinical UMN dysfunction was still observed in most ALSwoUMN. Regarding laboratory markers, serum cTnT levels tended to be higher in the ALSwoUMN group (24.0 [8.5–37.5] ng/L) than in the ALSwUMN group (12.0 [7.0–20.0] ng/L), although the difference did not reach statistical significance (*p* = 0.063). No significant differences were observed between the groups in FVC, serum creatinine, NfL, GFAP, or BDNF levels.

### 3.4 MRI Findings: Cortical Thickness and WM Volume

**FIGURE 3:**
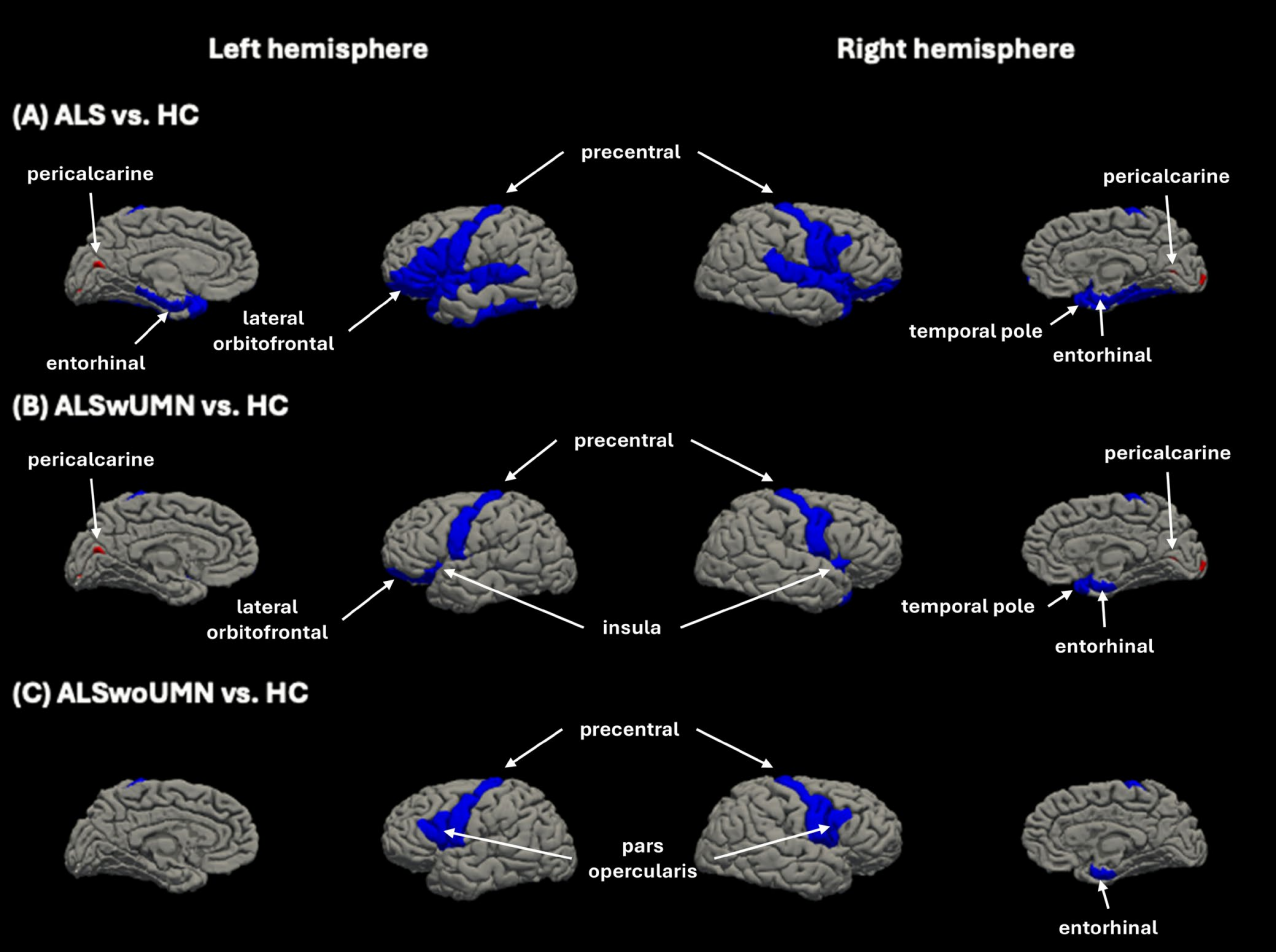
Comparison of cortical thickness. Cortical regions showing significant differences in the number of vertices between (A) ALS and healthy controls (HC; *p* ≤ 0.001), (B) ALSwUMN and HC (*p* < 0.05), and (C) ALSwoUMN and HC (*p* < 0.05). ALS, amyotrophic lateral sclerosis; ALSwoUMN, ALS patients without upper motor neuron signs; ALSwUMN, ALS patients with upper motor neuron signs; HC, healthy controls.

Figure 3 illustrates cortical thickness measurements across 37 bilateral cortical regions for each ALS group and controls. Table S2 and Figure S1 summarize cortical regions that showed significant differences in each patient group compared to controls. Both ALSwUMN and ALSwoUMN exhibited significant thinning of the bilateral precentral gyri and entorhinal cortex relative to healthy controls. ALSwUMN additionally showed significant atrophy in the lateral orbitofrontal cortex (primarily left‐sided) and temporal pole/insula (especially right‐sided). In contrast, ALSwoUMN demonstrated thinning restricted to the pars opercularis beyond the shared areas.

Interestingly, ALSwUMN exhibited subtle thickening of the pericalcarine cortex in both hemispheres, though this finding was not clinically relevant. Direct group‐wise comparison between ALSwUMN and ALSwoUMN revealed no significant differences after correction for multiple comparisons, although a qualitative trend toward more widespread cortical atrophy in ALSwUMN was noted.

**FIGURE 4:**
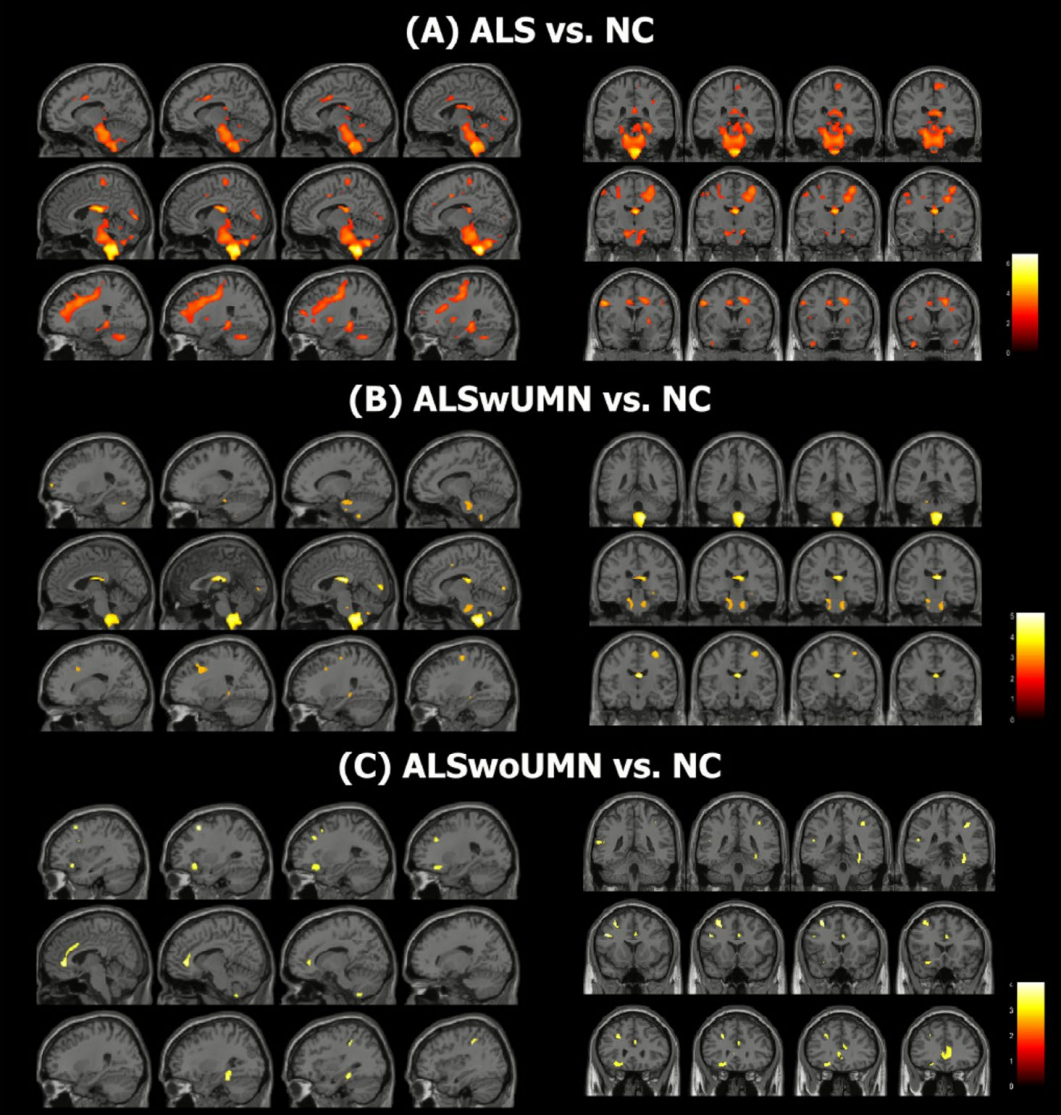
Comparison of white matter volume. Colored clusters indicate WM regions with significant volume differences between (A) ALS and HC, (B) ALSwUMN and HC, and (C) ALSwoUMN and HC (*p* < 0.001, uncorrected). ALS, amyotrophic lateral sclerosis; ALSwoUMN, ALS patients without upper motor neuron signs; ALSwUMN, ALS patients with upper motor neuron signs; HC, healthy controls; WM, white matter.

Regarding WM volume (Figure 4), ALSwUMN showed significant reductions compared with controls in the thalami, ventral diencephalon, cerebellar WM, amygdala, and brainstem (pons/medulla). ALSwoUMN demonstrated reductions in overlapping but fewer regions, specifically the thalami, right cerebellar WM, and left amygdala. Brainstem atrophy was observed only in ALSwUMN, suggesting greater involvement of corticospinal and corticobulbar tracts. Table S3 and Figure S2 summarize WM regions with significant atrophy in each group.

### 3.5 Survival Analysis

**FIGURE 5:**
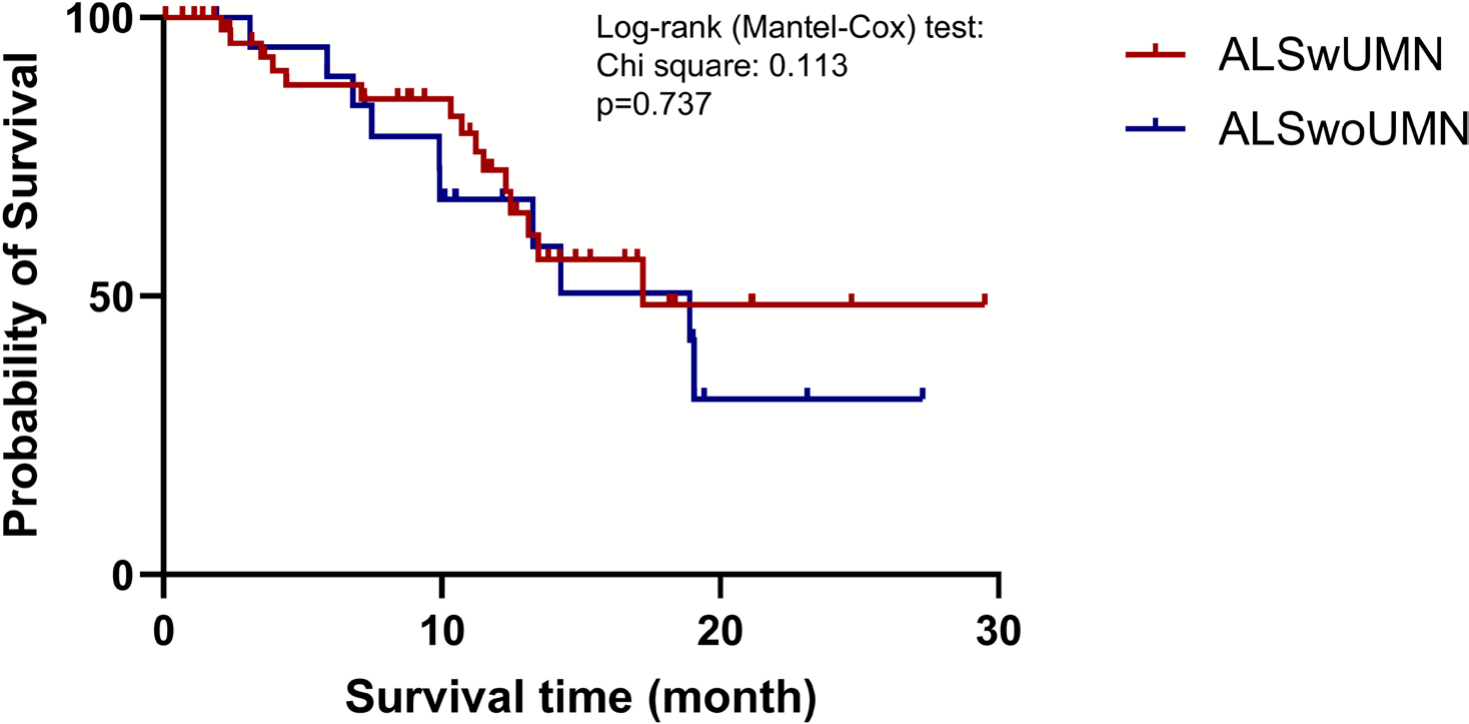
Kaplan–Meier survival curves comparing ALSwUMN and ALSwoUMN groups. ALS, amyotrophic lateral sclerosis; ALSwoUMN, ALS patients without upper motor neuron signs; ALSwUMN, ALS patients with upper motor neuron signs.

Kaplan–Meier analysis (Figure 5) showed no significant difference in overall survival between ALSwUMN and ALSwoUMN groups. In multivariate Cox proportional hazards regression (Table 2), only baseline disease progression rate was significantly associated with poorer survival (HR 2.091, 95% CI 1.339–3.265, *p* = 0.001). Other covariates, including age, sex, ALSFRS‐R score, site of symptom onset, and UMN status, were not significant predictors of survival.

**TABLE 2:** Multivariable Cox proportional hazards regression for survival.

	No. (*n* = 71)	Hazard ratio (95% CI)	*p*
Upper motor sign presence			
ALSwoUMN	20	1 (ref)	
ALSwUMN	51	0.917 (0.338, 2.486)	0.865
Sex			
Female	30	1 (ref)	
Male	41	1.345 (0.529, 3.419)	0.533
Age at onset	71	1.000 (0.957, 1.044)	0.984
ALSFRS‐R score	71	1.005 (0.953, 1.060)	0.847
Site of symptom onset			
Limb	49	1 (ref)	
Bulbar	22	1.007 (0.394, 2.571)	0.989
Progression rate at baseline	71	2.091 (1.339, 3.265)	0.001

Abbreviations: ALS, amyotrophic lateral sclerosis; ALSFRS‐R, ALS functional rating scale‐revised; ALSwoUMN, ALS patients without upper motor neuron signs; ALSwUMN, ALS patients with upper motor neuron signs; CI, confidence interval.

## 4 Discussion

This comparative study investigated whether ALSwoUMN differ meaningfully from ALSwUMN. Our findings show that ALSwoUMN largely share clinical features, disease trajectory, biomarker profiles, and neuroimaging abnormalities with ALSwUMN. These observations provide empirical support for the inclusive diagnostic approach of the Gold Coast criteria, which recognize ALSwoUMN as a valid presentation within the ALS spectrum.

As expected, electrophysiological assessments revealed a higher prevalence of abnormal MEPs in ALSwUMN, reflecting overt corticospinal tract dysfunction. Notably, a substantial proportion of ALSwoUMN also exhibited abnormal MEPs despite lacking clinical UMN signs. This suggests subclinical UMN involvement, corroborating prior studies using diffusion tensor imaging (DTI) and neuropathological analyses, which frequently detect corticospinal tract degeneration in patients previously diagnosed as PMA [14, 15, 24, 25]. These findings support the view that ALSwoUMN is not a distinct disease entity but rather a phenotypic variant along a continuum of ALS pathology. They also highlight the limitations of bedside neurological examination in detecting early or subtle UMN dysfunction.

Our imaging results reinforce this continuum. Both ALS groups showed cortical thinning in canonical ALS regions such as the precentral gyrus and entorhinal cortex, while ALSwUMN demonstrated broader involvement of the orbitofrontal cortex, insula, and parahippocampal areas. These findings align with prior MRI studies showing greater motor cortex atrophy in UMN‐predominant ALS [26, 27, 28, 29]. The selective thinning of the pars opercularis in ALSwoUMN is particularly intriguing and may reflect subtle differences in network vulnerability or routes of disease propagation. Recent work suggests that ALS pathophysiology involves large‐scale network degeneration beyond the motor system, and the distinct cortical patterns observed here may correspond to different trajectories of network degeneration [30, 31]. Similarly, WM analysis revealed shared degeneration of thalamic and cerebellar pathways in both groups, with additional brainstem involvement only in ALSwUMN. These results imply that although ALSwoUMN may initially present with more localized neurodegeneration, disease spread follows a similar trajectory over time.

The absence of significant differences in serum biomarkers (cTnT, NfL, GFAP, BDNF) suggests that these markers reflect the overall neurodegenerative process in ALS rather than specifically indicating the presence of clinical UMN signs. NfL, a cytoskeletal protein released during axonal degeneration, is markedly elevated in cerebrospinal fluid (CSF) or blood of patients with ALS [32, 33]. Although previous studies reported lower NfL levels in patients with purely LMN phenotypes [34, 35], our cohort exhibited substantial overlap between groups and no significant differences, supporting the concept of a shared pathogenic continuum between ALS with and without UMN signs.

Similarly, we analyzed serum cTnT, which has been suggested as an emerging marker in ALS [36, 37]. Our analysis showed a trend toward higher cTnT levels in the ALSwoUMN group compared with the ALSwUMN group (*p* = 0.063). Although this difference did not reach statistical significance, it may reflect recent insights into the source and relevance of cTnT elevation in ALS. Unlike the cardiac origin typically seen in myocardial injury, elevated cTnT in ALS—particularly in the absence of concomitant cardiac troponin I elevation—is increasingly considered to be of non‐cardiac origin, likely released from degenerating or regenerating skeletal muscle [36]. In addition, a recent study has demonstrated a direct correlation between serum cTnT levels and the extent of LMN involvement confirmed by electromyography [37]. From this perspective, the trend observed in our study—higher cTnT levels in ALSwoUMN—may further support its association with LMN burden, independent of UMN pathology. This supports the notion that cTnT could serve as a surrogate marker of denervation‐related skeletal muscle changes in ALS. However, given the lack of statistical significance, these findings should be interpreted cautiously, and further validation in larger cohorts is warranted.

GFAP, an intermediate filament protein expressed in astrocytes, serves as a marker of astrogliosis and neuroinflammation [38, 39]. Although not directly associated with motor decline, increased GFAP levels have been linked to cognitive impairment and shorter survival in ALS [40], suggesting it may reflect extramotor cortical involvement, such as frontotemporal dysfunction, rather than motor neuron degeneration alone. However, the lack of a significant difference in GFAP levels between the ALSwUMN and ALSwoUMN groups in our study suggests that astroglial activation is not tightly linked to the presence of clinical UMN signs.

BDNF, a neurotrophin essential for neuronal survival and synaptic plasticity, has been investigated as a potential biomarker in ALS [41, 42]. Dysregulation of BDNF and its precursor proBDNF has been associated with disease progression and survival, suggesting prognostic value [41]. However, in our cohort, BDNF levels did not differ significantly between patients with and without UMN signs, indicating no relationship between BDNF expression and UMN involvement.

Survival analysis further reinforces the clinical similarity between ALSwUMN and ALSwoUMN with no significant difference observed. Instead, disease progression rate emerged as the only independent predictor of poor outcome, consistent with previous studies emphasizing the prognostic value of functional decline [43]. In line with previous studies, our results support the view that LMN‐predominant phenotypes, including PMA, may not have a distinctly favorable prognosis [12, 44]. These findings hold important implications for both clinical care and research. Clinically, ALSwoUMN should receive the same level of monitoring and intervention as ALSwUMN. In research contexts, particularly clinical trials, exclusion of patients lacking clinical UMN signs may be unwarranted. Our results suggest that these patients experience comparable neurodegeneration and progression and may benefit equally from investigational therapies. As biomarkers and imaging techniques advanced, future studies may reveal molecular or genetic markers that further refine ALS subtypes classification and allow stratified therapeutic approaches [8, 9, 10, 45].

This study has several important limitations. Foremost, the modest sample size may have reduced the statistical power needed to detect subtle but potentially meaningful differences between groups. Although non‐significant, several findings in our dataset showed trends consistent with prior larger studies, suggesting that some associations might have emerged with a larger cohort. Furthermore, the single‐center design involving only Korean participants may limit the generalizability of our findings. In addition, although longitudinal outcomes such as survival were assessed, the follow‐up duration was relatively limited, which may have constrained our ability to detect long‐term divergences between the groups.

Accordingly, while our results provide preliminary support for the unified diagnostic approach of the Gold Coast criteria, they should be interpreted with caution and are not definitive. Large‐scale, multicenter longitudinal studies with extended follow‐up will be essential to validate these observations and to more clearly define the biological distinctions—if they exist—between ALS with and without clinical upper motor neuron signs.

## 5 Conclusion

ALS patients without clinical UMN signs exhibit clinical characteristics, biomarker profiles, neuroimaging abnormalities, and survival outcomes largely comparable to those with UMN signs. Although ALSwUMN display more overt corticospinal tract involvement, ALSwoUMN often show subclinical UMN pathology and do not constitute a separate disease entity. These findings affirm the Gold Coast criteria's unifying approach to ALS diagnosis, supporting the inclusion of ALSwoUMN within the ALS continuum. Future multicenter, longitudinal studies with larger cohorts are warranted to validate these results and further define the molecular and genetic landscape of ALS phenotypes.

## Author Contributions

Hee‐Jae Jung, E‐nae Cheong, Yangsean Choi, Hyunjin Kim, and Young‐Min Lim contributed to the conception and design of the study. Hee‐Jae Jung, E‐nae Cheong, Jungmin So, Heung‐Won Kang, Yangsean Choi, Eun‐Jae Lee, and Hyunjin Kim contributed to the acquisition and analysis of data. Hee‐Jae Jung, E‐nae Cheong, and Young‐Min Lim contributed to drafting the text or preparing the figures.

## Funding

This research was supported by grants from the Ministry of Science and ICT, South Korea (RS‐2023‐00211443); Asan Institute for Life Science, Asan Medical Center, Seoul, South Korea (2023IP0108).

## Conflicts of Interest

The authors declare no conflicts of interest.

## Supporting information


**Figure S1:** Mean cortical thickness showing significant differences among the groups.
**Figure S2:** Mean WM volumes showing significant differences among the groups.
**Table S1:** Characteristic comparison among the groups and covariate balance after full weighting.
**Table S2:** Mean cortical thickness showing significant differences among the groups.
**Table S3:** Mean WM volumes showing significant differences among the groups.

## Data Availability

The data supporting the findings of this study are available from the corresponding author upon reasonable request and with permission from the ethics committee.
