# Supplementary material for: ALS With and Without Upper Motor Neuron Signs: A Comparative Study Supporting the Gold Coast Criteria
Source: Ann Clin Transl Neurol. 2025 Dec 15;13(5):984–93. doi: 10.1002/acn3.70288 (PMC13161876; doi:10.1002/acn3.70288)
Supplement: Supplementary file 1 — Figure S1: Mean cortical thickness showing significant differences among the groups. Figure S2: Mean WM volumes showing significant differences among the groups. Table S1: Characteristic comparison among the groups and covariate balance after full weighting. Table S2: Mean cortical thickness showing significant differences among the groups. Table S3: Mean WM volumes showing significant differences among the groups. [file ACN3-13-984-s001.docx]

**Supplementary Materials**

**Supplementary Methods**

**Image analysis using FreeSurfer**

Cortical thicknesses were measured on 3D T1-weighted images using the FreeSurfer (version 7.3; http://surfer.nmr. mgh.harvard.edu). The technical details of these processes have been described in prior publications.^1,2^ In brief, the analysis included the following steps: (1) removal of nonbrain tissue (skull stripping); (2) inflation of the folded surface tessellation patterns; (3) automated Talairach transformation; (4) intensity normalization; (5) segmentation of gray matter (GM) and white matter (WM); and (6) tessellation of the GM/WM border and automated correction. A deformable surface algorithm was then used to obtain the GM/WM and gray matter/cerebrospinal fluid (CSF) surfaces. The cortical surface was parcellated into regions of interest (ROIs) using the Desikan-Killiany Atlas, from which average cortical thickness was calculated within each ROI. All images were visually inspected to identify any topological defects.³ Query, Design, Estimate, Contrast (QDEC), embedded within FreeSurfer, was used to statistically compare cortical thickness between groups, with a false discovery rate (FDR) threshold of 0.05 to control for multiple comparisons.

**Image analysis using SPM**

WM volumes were measured on 3D T1-weighted images using SPM12 (Wellcome Department of Imaging Neuroscience, London, United Kingdom). The MRI images were segmented into GM, WM, and CSF using a unified tissue-segmentation procedure that included correction for image intensity nonuniformity Segmentation was based on tissue probability maps aligned to the International Consortium for Brain Mapping (ICBM) template for East Asian brains. A study-specific mean GM and WM template was generated from individual images. These segmented GM and WM images were spatially normalized to this template using DARTEL (Diffeomorphic Anatomical Registration Through Exponentiated Lie Algebra).^4^ To preserve GM and WM volumes during spatial normalization, modulation was applied using Jacobian determinants derived from the DARTEL deformation fields. The modulated images were then smoothed with an 8-mm full width at half maximum (FWHM) Gaussian kernel.

Morphological group differences in these smoothed GM and WM images between amyotrophic lateral sclerosis (ALS) patients and healthy controls (HC) were analyzed using a two-sample *t*-test in SPM12. The same analysis was performed between the 42 ALS patients with upper motor neuron signs (ALSwUMN) and HC, and between the 16 ALS patients without upper motor neuron signs (ALSwoUMN) and HC. Group comparisons were assessed using a *p*-value threshold of < .001 (uncorrected, k > 100).

**References**

1. Fischl B, Dale AM. Measuring the thickness of the human cerebral cortex from magnetic resonance images. *Proceedings of the National Academy of Sciences*. 2000;97(20):11050-11055.

2. Fischl B, Salat DH, Busa E, et al. Whole brain segmentation: automated labeling of neuroanatomical structures in the human brain. *Neuron*. 2002;33(3):341-355.

3. Desikan RS, Ségonne F, Fischl B, et al. An automated labeling system for subdividing the human cerebral cortex on MRI scans into gyral based regions of interest. *Neuroimage*. 2006;31(3):968-980.

4. Ashburner J. A fast diffeomorphic image registration algorithm. *Neuroimage*. 2007;38(1):95-113.

**Supplementary Figure 1. Mean cortical thickness showing significant differences among the groups**


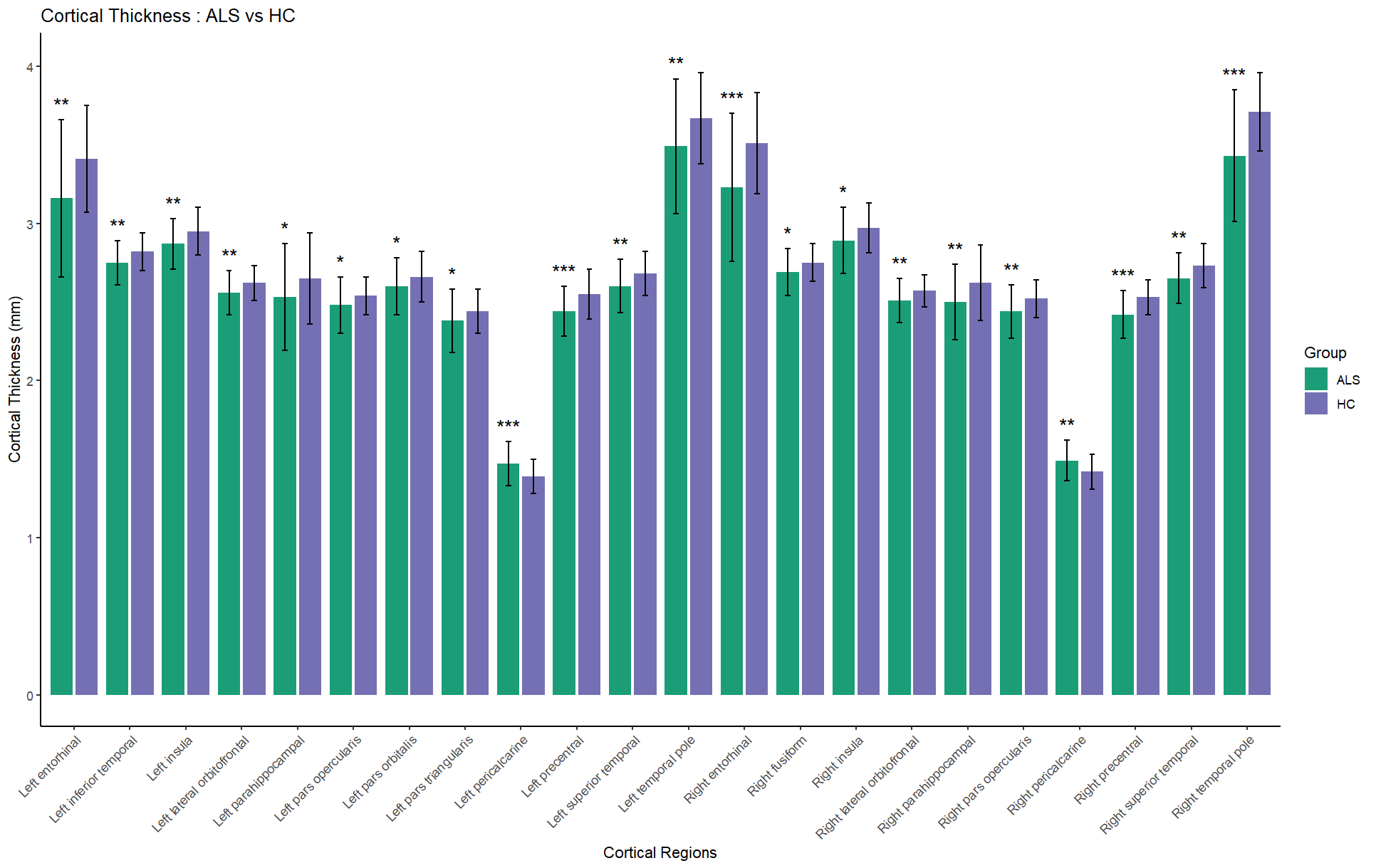


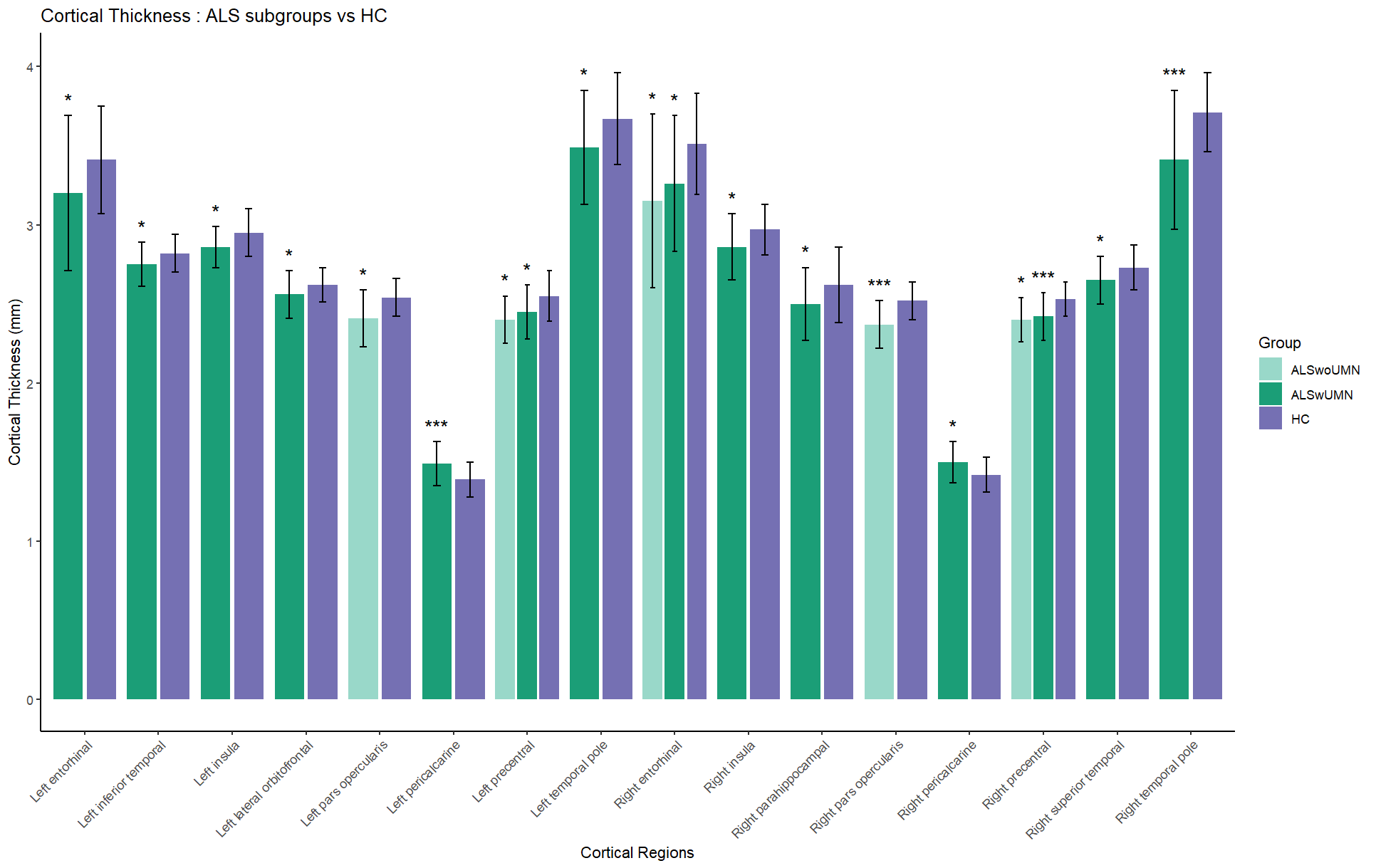


Top panel: Mean cortical thickness showing significant differences between ALS patients and HC. Bottom panel: Mean cortical thickness showing significant differences between ALS subgroups (ALSwoUMN, ALSwUMN) and HC.

ALS, amyotrophic lateral sclerosis; ALSwUMN, ALS patients with upper motor neuron signs; ALSwoUMN, ALS patients without upper motor neuron signs; HC, healthy controls

**Supplementary Figure 2. Mean WM volumes showing significant differences among the groups**


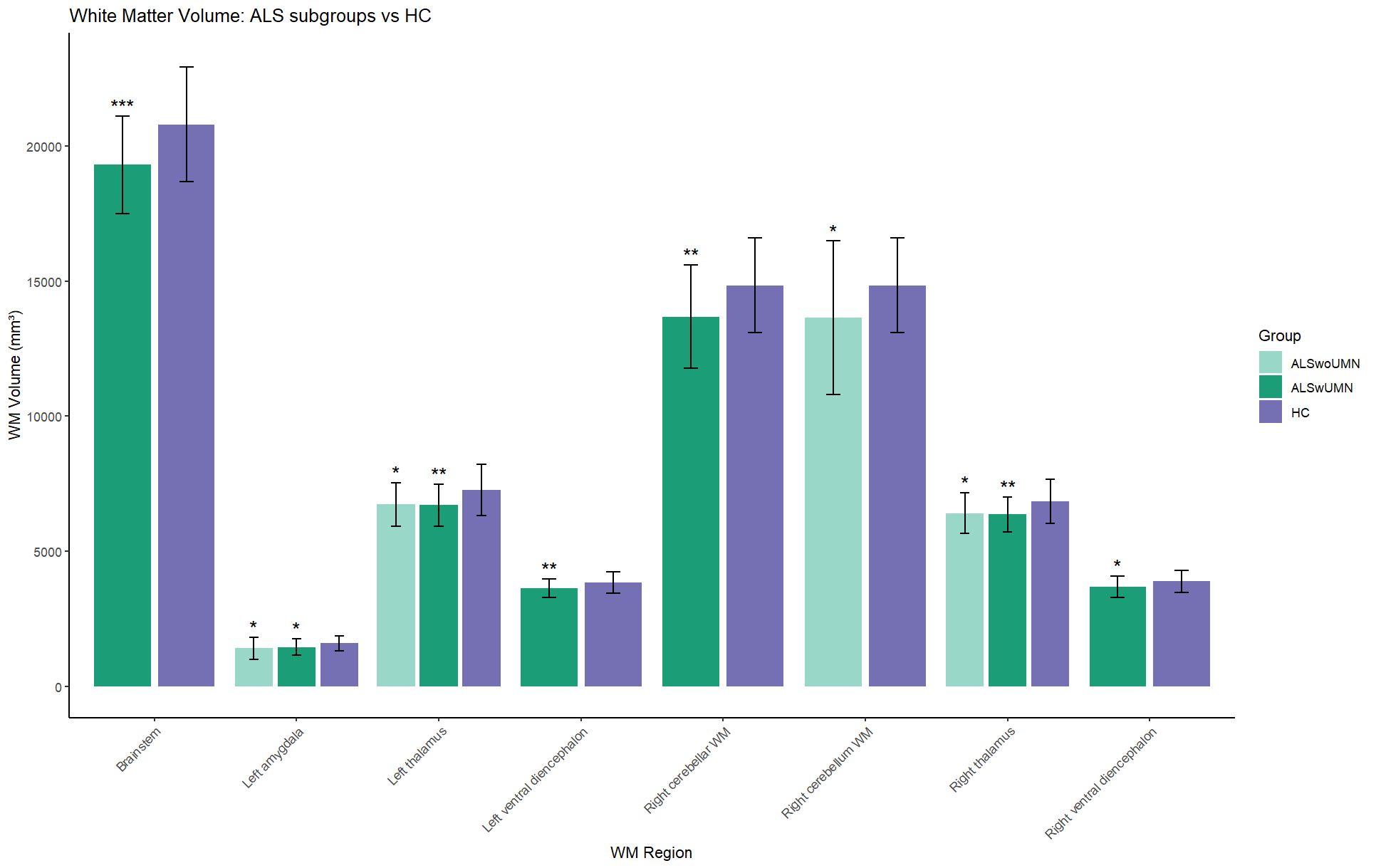


Mean WM showing significant differences between ALS subgroups (ALSwoUMN, ALSwUMN) and HC.

ALS, amyotrophic lateral sclerosis; ALSwUMN, ALS patients with upper motor neuron signs; ALSwoUMN, ALS patients without upper motor neuron signs; HC, healthy controls; WM, white matter

**Supplementary Table 1. Characteristic comparison among the groups and covariate balance after full weighting**

| Variable | HC (n=80) | ALS (n=58) | ALSwUMN (n=42) | ALSwoUMN (n=16) | p-value | SMD (after weighting) |
| --- | --- | --- | --- | --- | --- | --- |
| Distance |  |  |  |  |  | 0.024 |
| Age at enrollment, years | 61.3 ± 12.3 | 65.2 ± 11.0 | 63.9 ± 11.1 | 68.6 ± 10.2 | 0.054 / 0.24 / 0.019 | 0.008 |
| Male, n (%) | 45 (56.2%) | 41 (57.7%) | 24 (57.1%) | 12 (75%) | 0.609 / 1 / 0.2648 | 0.017 |

Note: p-value format: con vs Total ALS / con vs ALSwUMN / con vs ALSwoUMN, Distance row shows overall difference in propensity scores between groups

ALS, amyotrophic lateral sclerosis; ALSwUMN, ALS patients with upper motor neuron signs; ALSwoUMN, ALS patients without upper motor neuron signs; CI, confidence interval; HC, healthy controls; SMD, standardized mean difference after propensity score full weighting

**Supplementary Table 2. Mean cortical thickness showing significant differences among the groups**

| Cortical regions (mm) | ALS (n=58) | HC (n=80) | Mean difference [95% CI] | p-value |
| --- | --- | --- | --- | --- |
| Left pericalcarine | 1.47 ± 0.14 | 1.39 ± 0.11 | -0.08 [-0.12, -0.04] | **<0.001** |
| Left precentral | 2.44 ± 0.16 | 2.55 ± 0.16 | 0.113 [0.058, 0.167] | **<0.001** |
| Left entorhinal | 3.16 ± 0.50 | 3.41 ± 0.34 | 0.242 [0.101, 0.382] | **0.001** |
| Left lateral orbitofrontal | 2.56 ± 0.14 | 2.62 ± 0.11 | 0.067 [0.026, 0.107] | **0.001** |
| Left insula | 2.87 ± 0.16 | 2.95 ± 0.15 | 0.083 [0.030, 0.136] | 0.002 |
| Left superior temporal | 2.60 ± 0.17 | 2.68 ± 0.14 | 0.079 [0.027, 0.130] | 0.003 |
| Left inferior temporal | 2.75 ± 0.14 | 2.82 ± 0.12 | 0.067 [0.022, 0.111] | 0.003 |
| Left temporal pole | 3.49 ± 0.43 | 3.67 ± 0.29 | 0.176 [0.056, 0.295] | 0.004 |
| Left pars opercularis | 2.48 ± 0.18 | 2.54 ± 0.12 | 0.060 [0.010, 0.109] | 0.017 |
| Left parahippocampal | 2.53 ± 0.34 | 2.65 ± 0.29 | 0.114 [0.008, 0.219] | 0.034 |
| Left pars triangularis | 2.38 ± 0.20 | 2.44 ± 0.14 | 0.062 [0.004, 0.119] | 0.035 |
| Left pars orbitalis | 2.60 ± 0.18 | 2.66 ± 0.16 | 0.060 [0.002, 0.118] | 0.042 |
| Right precentral | 2.42 ± 0.15 | 2.53 ± 0.11 | 0.111 [0.067, 0.155] | **<0.001** |
| Right temporal pole | 3.43 ± 0.42 | 3.71 ± 0.25 | 0.276 [0.164, 0.388] | **<0.001** |
| Right entorhinal | 3.23 ± 0.47 | 3.51 ± 0.32 | 0.276 [0.145, 0.407] | **<0.001** |
| Right pericalcarine | 1.49 ± 0.13 | 1.42 ± 0.11 | -0.06 [-0.11, -0.02] | **0.001** |
| Right pars opercularis | 2.44 ± 0.17 | 2.52 ± 0.12 | 0.080 [0.031, 0.130] | 0.002 |
| Right superior temporal | 2.65 ± 0.16 | 2.73 ± 0.14 | 0.076 [0.026, 0.125] | 0.003 |
| Right parahippocampal | 2.50 ± 0.24 | 2.62 ± 0.24 | 0.123 [0.041, 0.205] | 0.003 |
| Right lateral orbitofrontal | 2.51 ± 0.14 | 2.57 ± 0.10 | 0.061 [0.020, 0.101] | 0.004 |
| Right insula | 2.89 ± 0.21 | 2.97 ± 0.16 | 0.078 [0.016, 0.141] | 0.013 |
| Right fusiform | 2.69 ± 0.15 | 2.75 ± 0.12 | 0.052 [0.006, 0.097] | 0.025 |

| **Cortical regions (mm)** | **ALSwUMN (n=42)** | **HC (n=80)** | **Mean difference [95% CI]** | ***p*-value** |
| --- | --- | --- | --- | --- |
| Left pericalcarine | 1.49 ± 0.14 | 1.39 ± 0.11 | -0.102 [-0.15, -0.05] | **<0.001** |
| Left precentral | 2.45 ± 0.17 | 2.55 ± 0.16 | 0.100 [0.04, 0.16] | **0.012** |
| Left insula | 2.86 ± 0.13 | 2.95 ± 0.15 | 0.094 [0.033, 0.156] | **0.02** |
| Left lateral orbitofrontal | 2.56 ± 0.15 | 2.62 ± 0.11 | 0.064 [0.019, 0.11] | **0.047** |
| Left entorhinal | 3.20 ± 0.49 | 3.41 ± 0.34 | 0.209 [0.054, 0.364] | 0.05 |
| Left inferior temporal | 2.75 ± 0.14 | 2.82 ± 0.12 | 0.068 [0.016, 0.12] | 0.05 |
| Left temporal pole | 3.49 ± 0.36 | 3.67 ± 0.29 | 0.184 [0.044, 0.323] | 0.05 |
| Right precentral | 2.42 ± 0.15 | 2.53 ± 0.11 | 0.106 [0.057, 0.154] | **<0.001** |
| Right temporal pole | 3.41 ± 0.44 | 3.71 ± 0.25 | 0.297 [0.167, 0.428] | **<0.001** |
| Right entorhinal | 3.26 ± 0.43 | 3.51 ± 0.32 | 0.246 [0.101, 0.391] | **0.012** |
| Right pericalcarine | 1.50 ± 0.13 | 1.42 ± 0.11 | -0.078 [-0.125, -0.031] | **0.012** |
| Right insula | 2.86 ± 0.21 | 2.97 ± 0.16 | 0.101 [0.029, 0.173] | **0.047** |
| Right parahippocampal | 2.50 ± 0.23 | 2.62 ± 0.24 | 0.126 [0.03, 0.222] | 0.05 |
| Right superior temporal | 2.65 ± 0.15 | 2.73 ± 0.14 | 0.075 [0.02, 0.13] | 0.05 |
| **Cortical regions (mm)** | **ALSwoUMN (n=16)** | **HC (n=80)** | **Mean difference [95% CI]** | ***p*-value** |
| Left precentral | 2.40 ± 0.15 | 2.55 ± 0.16 | 0.148 [0.056, 0.239] | **0.018** |
| Left pars opercularis | 2.41 ± 0.18 | 2.54 ± 0.12 | 0.126 [0.044, 0.208] | **0.028** |
| Right pars opercularis | 2.37 ± 0.15 | 2.52 ± 0.12 | 0.150 [0.068, 0.232] | **<0.001** |
| Right entorhinal | 3.15 ± 0.55 | 3.51 ± 0.32 | 0.358 [0.139, 0.578] | **0.018** |
| Right precentral | 2.40 ± 0.14 | 2.53 ± 0.11 | 0.127 [0.054, 0.200] | **0.018** |

ALS, amyotrophic lateral sclerosis; ALSwUMN, ALS patients with upper motor neuron signs; ALSwoUMN, ALS patients without upper motor neuron signs; CI, confidence interval; HC, healthy controls

**Supplementary Table 3.** **Mean WM volumes showing significant differences among the groups**

| **WM regions (mm^3^)** | **ALSwUMN (n=42)** | **HC (n=80)** | **Mean difference [95% CI]** | ***p*-value** |
| --- | --- | --- | --- | --- |
| Brainstem | 19293.5 ± 1803.1 | 20790 ± 2125.5 | 1496.4 [696.0, 2296.9] | <0.001 |
| Right thalamus | 6366.28 ± 647.96 | 6859.10 ± 817.00 | 492.8 [191.7, 794.0] | 0.001 |
| Left thalamus | 6713.00 ±778.05 | 7275.24 ± 952.40 | 562.2 [211.9, 912.6] | 0.002 |
| Right cerebellar WM | 13682.31 ± 1912.20 | 14838.11 ± 1746.93 | 1155.8 [426.5, 1885.1] | 0.002 |
| Left ventral diencephalon | 3642.59 ± 347.46 | 3857.52 ± 391.51 | 214.9 [63.1, 366.8] | 0.005 |
| Right ventral diencephalon | 3689.03 ± 390.63 | 3890.14 ± 397.81 | 201.1 [46.2, 356.0] | 0.011 |
| Left amygdala | 1456.64 ± 304.75 | 1599.22 ± 271.89 | 142.6 [30.2, 255.0] | 0.013 |
| **WM regions (mm^3^)** | **ALSwoUMN (n=16)** | **HC (n=80)** | **Mean difference [95% CI]** | ***p*-value** |
| Left thalamus | 6732.10 ± 797.65 | 7275.24 ± 952.40 | 543.1 [63.7, 1022.6] | 0.027 |
| Right thalamus | 6413.37 ± 748.29 | 6859.10 ± 817.00 | 445.7 [33.6, 857.9] | 0.034 |
| Right cerebellum WM | 13645.64 ± 2833.22 | 14838.11 ± 1746.93 | 1192.5 [86.9, 2298.0] | 0.034 |
| Left amygdala | 1417.06 ± 407.93 | 1599.22 ± 271.89 | 182.2 [11.8, 352.5] | 0.036 |

ALS, amyotrophic lateral sclerosis; ALSwUMN, ALS patients with upper motor neuron signs; ALSwoUMN, ALS patients without upper motor neuron signs; CI, confidence interval; HC, healthy controls; WM, white matter
